# Passive acoustic monitoring of killer whales (*Orcinus orca*) reveals year-round distribution and residency patterns in the Gulf of Alaska

**DOI:** 10.1038/s41598-021-99668-0

**Published:** 2021-10-13

**Authors:** Hannah J. Myers, Daniel W. Olsen, Craig O. Matkin, Lara A. Horstmann, Brenda Konar

**Affiliations:** 1 College of Fisheries and Ocean Sciences, University of Alaska Fairbanks, 2150 Koyukuk Dr., Fairbanks, AK 99775 USA; 2 North Gulf Oceanic Society, 3430 Main St., Suite B1, Homer, AK 99603 USA

**Keywords:** Marine mammals, Conservation biology, Marine biology

## Abstract

Killer whales (*Orcinus orca*) are top predators throughout the world’s oceans. In the North Pacific, the species is divided into three ecotypes—resident (fish-eating), transient (mammal-eating), and offshore (largely shark-eating)—that are genetically and acoustically distinct and have unique roles in the marine ecosystem. In this study, we examined the year-round distribution of killer whales in the northern Gulf of Alaska from 2016 to 2020 using passive acoustic monitoring. We further described the daily acoustic residency patterns of three killer whale populations (southern Alaska residents, Gulf of Alaska transients, and AT1 transients) for one year of these data. Highest year-round acoustic presence occurred in Montague Strait, with strong seasonal patterns in Hinchinbrook Entrance and Resurrection Bay. Daily acoustic residency times for the southern Alaska residents paralleled seasonal distribution patterns. The majority of Gulf of Alaska transient detections occurred in Hinchinbrook Entrance in spring. The depleted AT1 transient killer whale population was most often identified in Montague Strait. Passive acoustic monitoring revealed that both resident and transient killer whales used these areas much more extensively than previously known and provided novel insights into high use locations and times for each population. These results may be driven by seasonal foraging opportunities and social factors and have management implications for this species.

## Introduction

Killer whales (*Orcinus orca*) are cosmopolitan top predators, with a minimum global population estimate of 50,000 animals found throughout all of the world’s oceans^1^. In the North Pacific, the species has diverged into three genetically distinct, sympatric ecotypes that display different dietary habitats, vocal behavior, and social structure^2–5^. Resident killer whales consume primarily fish, especially salmonids, and live in stable social units of maternally related whales^6–11^. Transient killer whales prey on marine mammals and are typically observed in smaller, more fluid social groups^7,9,12,13^. Offshore killer whales are less well-studied, but appear to specialize on sharks and have a primarily pelagic distribution^14–16^. The three ecotypes may be considered separate species, as transients diverged from other lineages an estimated 250,000 to 350,000 years ago and residents and offshores diverged from each other at least 50,000 years ago^17–19^.

Killer whale vocalizations have been categorized into three functionally distinct groups: clicks, pulsed calls, and whistles^20–25^. Clicks are used primarily for echolocation, especially while foraging^25,26^. Pulsed calls have high pulse-repetition rates that result in tonal sounds rich with harmonic structure, and are thought to be used for behavioral coordination and group recognition^25,27–30^. Whistles are tonal sounds with minimal harmonic structure and have been documented primarily when animals are socializing in close proximity^25,31,32^.

Resident, transient, and offshore killer whales all utilize these three vocalization types, but differ in aspects of their vocal behavior. Both resident and offshore killer whales are highly vocal, and commonly vocalize while foraging, socializing, and traveling^16,25–27^. Both of these ecotypes also commonly utilize echolocation clicks while foraging^16,25,26^. In contrast, transient killer whales have rarely been recorded vocalizing except during and after a kill^32–34^. To avoid detection by their marine mammal prey, which generally have sensitive underwater hearing within the frequency range of killer whale vocalizations, transients utilize a foraging strategy of stealth, acoustic crypsis, and passive listening^26,33,35^. The ecotypes also vary in their call pitch, with offshores utilizing the highest frequencies, followed by residents and then transients^36^.

Different killer whale populations produce unique repeated pulsed calls, called “discrete” or “stereotyped” calls^27,33,34,37,38^. Discrete calls can be reliably distinguished aurally and visually on a spectrogram and used to differentiate among populations^37,39–42^. Transient killer whale calls are also often characterized by an audible quavering of the fundamental sound frequencies and fewer call syllables^40,43^. Repertoires of discrete calls, known as dialects, can be further used to distinguish between resident killer whale pods, which are groups of related killer whales that typically swim together^11,27,37,38^. The degree of similarity across dialects reflects the genetic relatedness of different groups^4,37^.

Four populations of killer whales from three ecotypes inhabit the northern Gulf of Alaska. The southern Alaska resident population likely includes over 1000 animals, with 33 documented pods^44,45^. This population has increased consistently over the last several decades at an approximately 3.4% annual growth rate^44^. The southern Alaska residents range from waters off Kodiak Island to southeast Alaska^3,46–48^. The Gulf of Alaska (GOA) transient population ranges from Kodiak Island to southeast Alaska, and likely includes over 100 animals^47,49^. The GOA transients are rarely sighted, but the population appears stable^45,49^. The AT1 transients are a genetically distinct population of killer whales that primarily inhabit Prince William Sound and Kenai Fjords^4,49^. Originally, in 1984, twenty-two animals were documented in this population, which is hypothesized to be a remnant group of a once larger transient population^4,34,50^. Following the *Exxon Valdez* oil spill, the AT1 transients immediately lost nine members, and the population has since declined to seven animals and is expected to become extinct^50^. The offshore killer whale population is less well-studied, but is estimated to number over 300 animals and has been sighted from the Aleutian Islands to southern California^14,16^.

Previous studies using vessel surveys and satellite telemetry have described the summertime distribution of killer whales in the northern Gulf of Alaska^3,9,46,48,49,51,52^. However, vessel surveys were limited by season, weather, and daylight hours, while satellite telemetry was limited by the duration of tag attachment (typically fewer than 25 days), expense, and animal welfare concerns^48,52^. The vast majority of previous observations of killer whales in this region occurred between May and October. Understanding the year-round spatiotemporal distribution of killer whales is important to evaluate how each of these top predator populations affects ecological communities in the northern Gulf of Alaska. This is especially valuable in the face of widespread loss of apex consumers and subsequent trophic cascades^53^, as well as unprecedented climate change that may fundamentally alter many marine ecosystems^54,55^. All killer whales in U.S. waters are also federally protected under the Marine Mammal Protection Act. Research on their distribution and habitat use patterns is important to inform effective management policy^56^.

Passive acoustic monitoring is an effective, non-invasive method to continuously monitor cetaceans year-round in specific areas (e.g.,^57–59^, including to study killer whales^60–63^. In this study, we examined how killer whale spatiotemporal distribution patterns change throughout the year in the northern GOA. Additionally, we asked how daily acoustic residency patterns differ for the southern Alaska residents, GOA transients, and AT1 transients.

## Results

### Seasonal detection patterns

**Figure 1:**
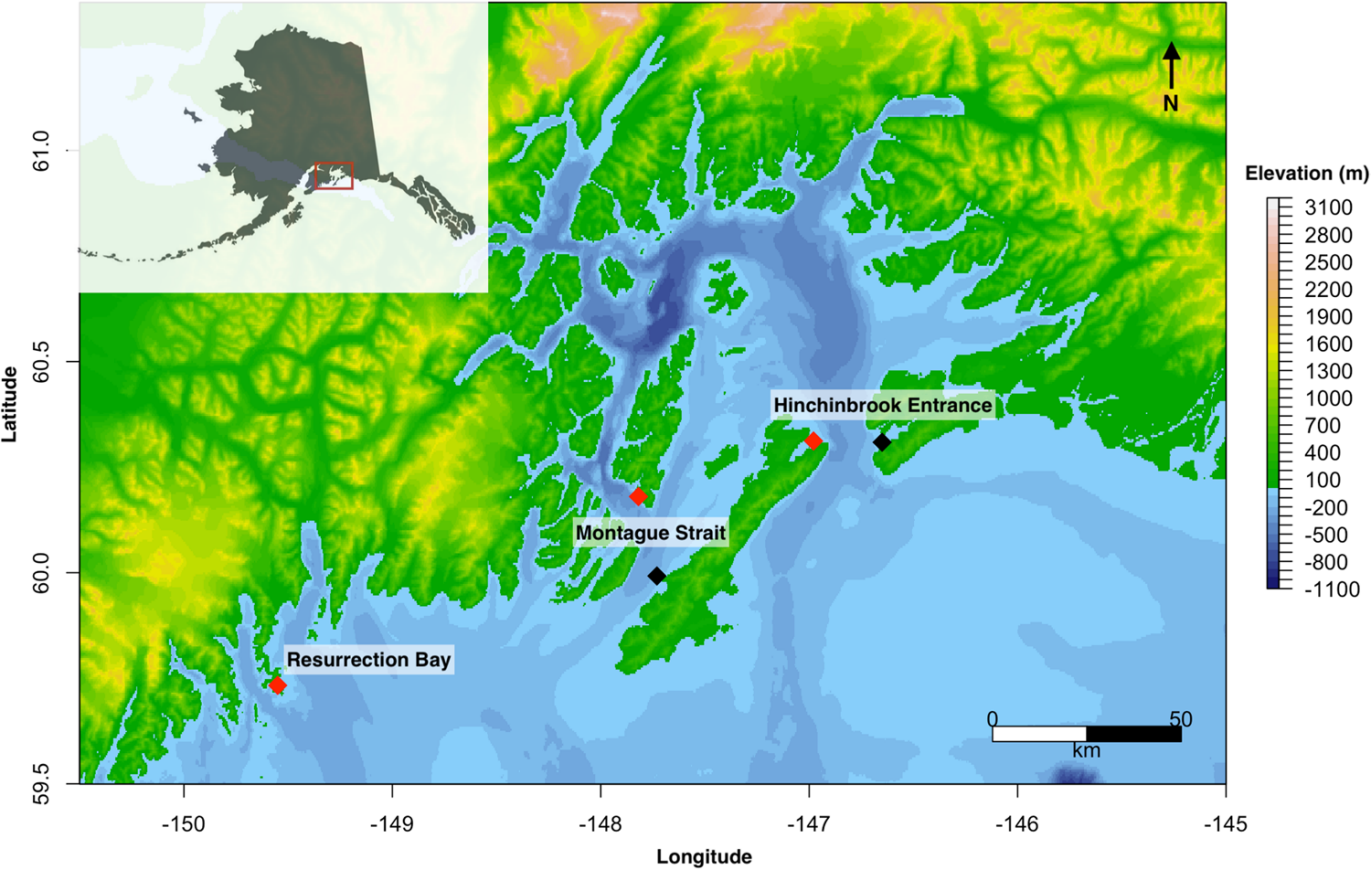
Map of hydrophone locations in the northern Gulf of Alaska. Red points indicate final hydrophone sites in Resurrection Bay, Montague Strait (Little Bay), and Hinchinbrook Entrance (Zaikof Bay). Black points indicate initial hydrophone sites in Montague Strait (Hanning Bay) and Hinchinbrook Entrance (Port Etches). Inset map shows location of study area in the northern Gulf of Alaska.

**Figure 2:**
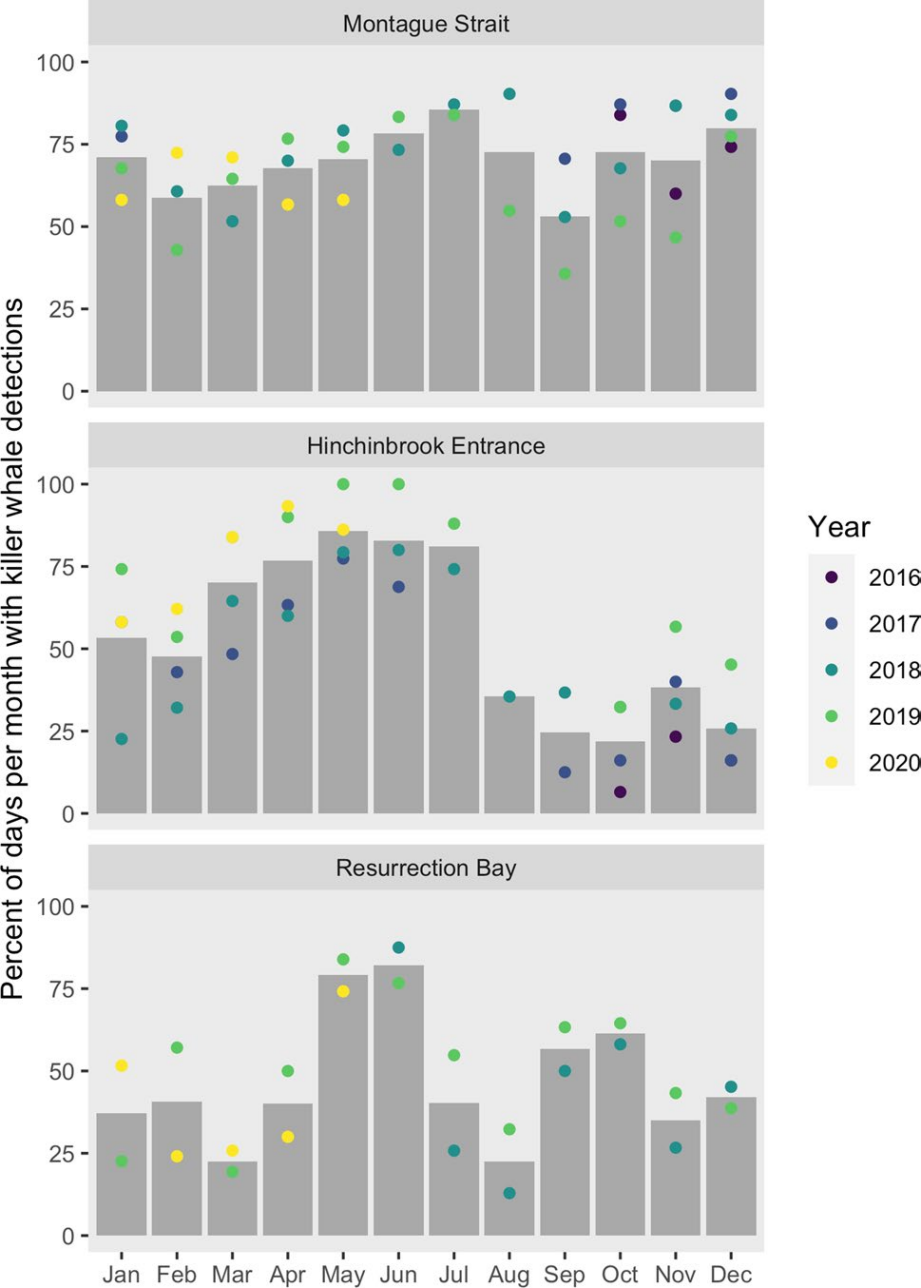
Percent of days per month that killer whales were detected at Montague Strait, Hinchinbrook Entrance, and Resurrection Bay, Gulf of Alaska, October 2016 to May 2020. Columns represent the average across years at each location, colored points represent values for each year recordings were available.

Killer whales were detected on 58.0% (1743 of 3003) of recording days across the three locations: Montague Strait, Hinchinbrook Entrance, and Resurrection Bay (Fig. 1). Killer whales were detected most consistently throughout the year in Montague Strait (Fig. 2). Overall, killer whales were detected on 70.1% (766 of 1092) of days recorded at Montague Strait (SD = 14.4%, range = 35.7% to 90.3% per month). A binomial logistic regression (*n* = 1092) showed that the percent of days with killer whales was significantly higher than the mean in July (85.5%, *p* = 0.0121) and December (79.8%, *p* = 0.0253), and lower than the mean in February (58.7%, *p* = 0.0306) and September (53.1%, *p* = 0.0011). The detection pattern was consistent across the Hanning Bay and Little Bay hydrophone sites.

In Hinchinbrook Entrance, there was a strong seasonal pattern of killer whale detections (Fig. 2). Killer whales were detected 54.1% (642 of 1186) of days recorded at Hinchinbrook Entrance (SD = 26.9%, range = 6.5% to 100% per month). A binomial logistic regression (*n* = 1186) showed that acoustic presence was significantly higher than the mean from March to July (March: 70.2%, *p* = 0.0008; April: 76.7%, *p* < 0.0001; May: 85.7%, *p* < 0.0001; June: 82.9%, *p* < 0.0001; July: 81.1%, *p* = 0.0003) and lower than the mean from August to December (August: 35.5%, *p* = 0.0445; September: 24.6%, *p* = 0.0001; October: 21.8%, *p* < 0.0001; November: 38.3%, *p* = 0.0011; December: 25.8%, *p* < 0.0001). This seasonal pattern was consistent across the Port Etches and Zaikof Bay hydrophone sites, though monthly detection rates were higher during the Zaikof Bay deployment period.

There was also a strong seasonal pattern of killer whale detections at Resurrection Bay (Fig. 2). Killer whales were detected on 46.2% (335 of 725) of days recorded at Resurrection Bay (SD = 21.3%, range = 12.9% to 87.5% per month). Binomial logistic regression (*n* = 725) results showed that killer whales were detected significantly more often than the mean in May, June, and October (May: 79.1%, *p* < 0.0001; June: 82.1%, *p* < 0.0001; October: 61.3%, *p* = 0.0241). Detections were significantly lower than the mean in March and August (both 22.6%, *p* = 0.0006).

### Daily residency times by population

Between June 2019 and May 2020, killer whales were detected in 7090 recordings (a recording is defined as the four or five minute “on” period of a 15 min or 20 min duty cycle). Southern Alaska resident calls were present in 92.1% of these recordings, Gulf of Alaska transients were detected in 2.7%, AT1 transients were detected in 1.6%, and 4.3% of recordings with killer whale detections included no calls that could be attributed to a population. Offshore killer whales were detected on one day (April 17th, 2020) in Resurrection Bay.

**Figure 3:**
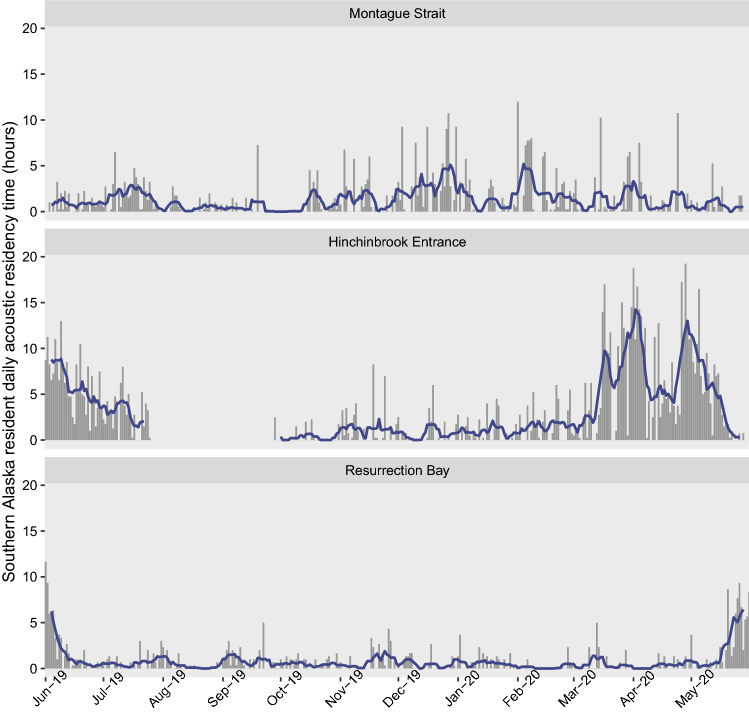
Hours per day that southern Alaska resident killer whales were detected in Montague Strait, Hinchinbrook Entrance, and Resurrection Bay, Gulf of Alaska, June 2019 to May 2020. Gray bars represent daily hours with detections, blue line is a weekly rolling average. No data were available at Hinchinbrook Entrance from July 26th–September 27th, 2019 and May 30th–31st, 2020 and at Montague Strait from September 25th–26th, 2019.

The number of hours per day (calculated as the number of recordings with detections divided by four for 15 min duty cycles and divided by three for 20 min duty cycles) that southern Alaska resident killer whales were detected at each location paralleled seasonal detection patterns. In Montague Strait, resident killer whales were consistently detected for multiple hours per day throughout the year (Fig. 3). In Hinchinbrook Entrance, resident killer whales were detected for the greatest number of hours per day from March to July, with peaks in mid-March, late March/early April, and late April/early May (Fig. 3). The acoustic residency time of resident killer whales during these periods—up to 19.25 h per day—was substantially higher than during any other time or location (Fig. 3). In Resurrection Bay, resident killer whales were detected for the greatest number of hours per day during May and June (up to 11.67 h), but high acoustic presence in October, as measured by percent of days per month, was not reflected in daily acoustic residency time (Fig. 3).

**Figure 4:**
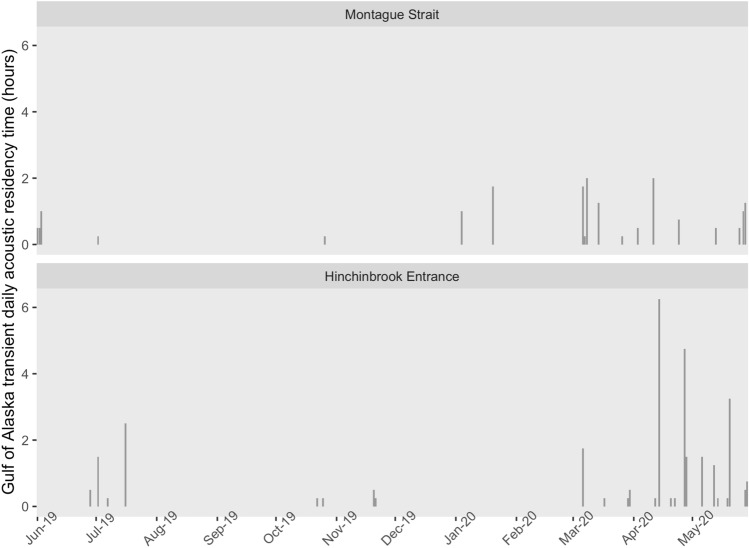
Hours per day with Gulf of Alaska transient killer whale detections in Montague Strait and Hinchinbrook Entrance, Gulf of Alaska, June 2019 to May 2020.

GOA transients were recorded on 41 days, including 25 days in Hinchinbrook Entrance and 19 days in Montague Strait (Fig. 4). GOA transients were detected in both Hinchinbrook Entrance and Montague Strait on three days. They were not detected in Resurrection Bay between June 2019 and May 2020. GOA transients and southern Alaska residents were both acoustically present in 32 recordings and GOA transients were twice detected in the same recording as AT1 transients, all of which occurred from March to May 2020 in Hinchinbrook Entrance. Of all of the recordings of GOA transients across locations, 60.6% (114 of 188) took place in Hinchinbrook Entrance between March and July, a period that coincides with the highest monthly detection rates and daily acoustic residency times for resident killer whales. GOA transients were recorded for up to 6.25 h in a single day (Fig. 4).

**Figure 5:**
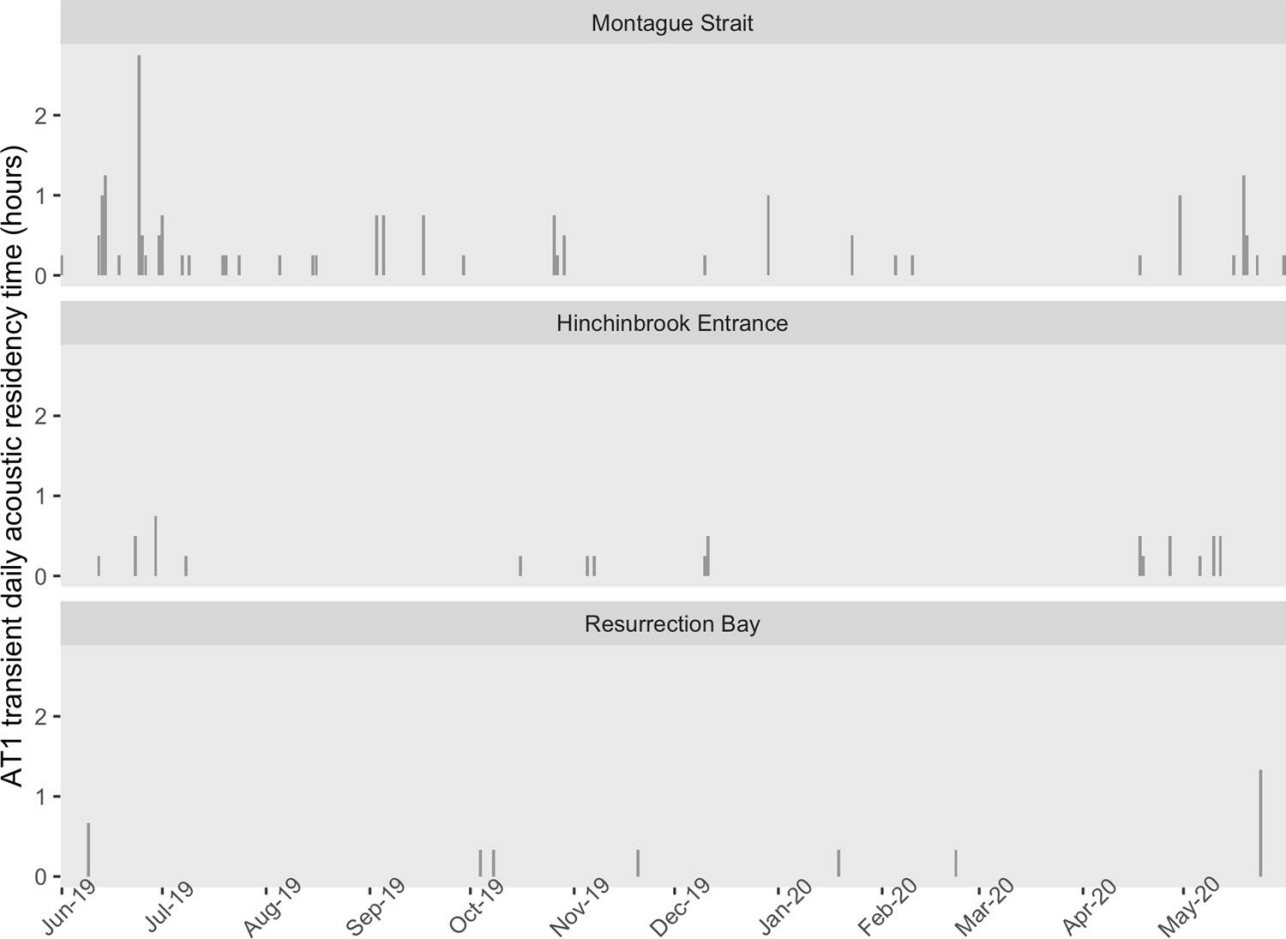
Hours per day with AT1 transient killer whale detections at Montague Strait, Hinchinbrook Entrance, and Resurrection Bay, Gulf of Alaska, June 2019 to May 2020.

AT1 transient killer whales were recorded on 56 days, including 37 days in Montague Strait, 15 days in Hinchinbrook Entrance, and 7 days in Resurrection Bay (Fig. 5). AT1s were detected in both Montague Strait and Hinchinbrook Entrance on three days. AT1 transients and southern Alaska residents were both detected in 14 recordings, the majority of which (10 of 14) occurred in June 2019 and April and May 2020 in Hinchinbrook Entrance. AT1s were detected for up to 2.75 h in a single day (Fig. 5).

### Duty cycle

There were a total of 7090 recordings with killer whale detections from June 2019 to May 2020. Overall, 82.1% (5830 recordings) included killer whale detections in the first half of the recording. The percentage of recordings with killer whales in the first half was very similar across locations (Resurrection Bay: 81.8%, Montague Strait 81.3%, Hinchinbrook Entrance 82.6%) (Table 1). These locations recorded on different duty cycles during this time period. Resurrection Bay recorded 4 min on, 16 min off (4/20 min); Montague Strait recorded 5 min on, 10 min off (5/15 min); and Hinchinbrook Entrance recorded 4 min on, 11 min off (4/15 min) for two months and 5/15 min for eight months. These results demonstrate that 2/20 min, 2/15 min, and 2.5/15 min duty cycles are comparably effective in detecting acoustic presence, and > 80% as effective as duty cycles with twice as much recording time. Therefore, we did not apply a correction factor to compare data collected on different duty cycles.

**Table 1:** Percentage of recordings with killer whale detections at hydrophone locations in the northern Gulf of Alaska in which vocalizations were present in the first half of the recording.

Month-year	Montague strait	Hinchinbrook entrance	Resurrection bay
Jun-2019	73.6	78.2^†^	84.6*
Jul-2019	76.2	76.3^†^	83.6*
Aug-2019	76.9	N/A	70.4*
Sep-2019	76.7	N/A	77.2*
Oct-2019	68.4	75.6	78.8*
Nov-2019	81.6	79.3	77.8*
Dec-2019	86.5	71.6	78.3*
Jan-2020	77.3	74.6	87.3*
Feb-2020	86.1	74.6	82.4*
Mar-2020	86.4	86.0	80.9*
Apr-2020	86.4	89.7	79.3*
May-2020	73.5	82.8	83.5*
Total	81.3	82.6	81.8*

Months that are not otherwise denoted recorded on a 5 min on, 10 min off duty cycle.

*4 min on, 16 min off duty cycle.

^†^4 min on, 11 min off duty cycle.

Binomial logistic regression (*n* = 7090) results indicated that the probability of detecting killer whales using a shorter duty cycle was significantly higher in March (85.9%, *p* = 0.0062) and April (89.0%, *p* = 0.0001) than the rest of the year (Table 1). This difference was driven by the high number of recordings with killer whale vocalizations in the first half at Hinchinbrook Entrance during March (615 recordings) and April (909 recordings). Because this difference was localized, a seasonal correction factor was not applied across locations.

Of 6536 recordings of resident killer whales, 85.5% (5589 recordings) included vocalizations in the first half of the recording. Of 188 recordings of GOA transients, 84.6% (159 recordings) had vocalizations in the first half. In contrast, only 57.9% (66 of 114) of recordings of AT1 transients had vocalizations in the first half.

## Discussion

This study provides the first description of year-round killer whale presence in the northern Gulf of Alaska. We found significant differences in acoustic presence throughout the year and differences among locations. Daily acoustic residency times differed among the southern Alaska residents, GOA transients, and AT1 transients, likely reflecting the unique role each population plays in these areas as well as probable differences in vocal behavior. Passive acoustic monitoring revealed that resident and transient killer whales used these areas much more extensively than previously thought and provided new insight into timing and location of use for each killer whale population. These patterns may be driven by seasonal foraging opportunities and social factors.

This study particularly highlights the importance of Montague Strait for southern Alaska resident killer whales in winter months, a previously unstudied period. Resident killer whales were acoustically present in Montague Strait for up to twelve hours per day in winter months, and daily acoustic residency times were greater during winter than summer months. In Montague Strait, high acoustic presence throughout the year might suggest that there are consistent suitable foraging opportunities in this area. Research conducted from May to October indicates that the southern Alaska resident population feeds primarily upon Chinook (*Oncorhynchus tshawytscha*), coho (*Oncorhynchus kisutch*), and chum (*Oncorhynchus keta*) salmon, but they have also been documented foraging on Pacific halibut (*Hippoglossus stenolepis*), arrowtooth flounder (*Atheresthes stomias*), sockeye salmon (*Oncorhynchus nerka*), and Pacific herring (*Clupea pallasii*), and depredating on sablefish (*Anoplopoma fimbria*) longlines (^9,45,64^, NGOS unpublished data). Fish scales collected from predation sites indicate that southern Alaska residents preferentially consume Chinook and chum salmon in spring, followed by chum and coho salmon in summer and fall^9,45^. The southern resident killer whales, a population of residents that inhabits the Salish Sea and waters off the western United States, also consume almost exclusively salmonids—especially Chinook salmon—from May to September^65^. However, the southern residents diversify their diet to include other salmonid and non-salmonid species in fall and winter when Chinook salmon are less available^66^. Similarly, in fall, winter, and early spring, southern Alaska resident killer whales may consume any available salmon, but also forage on species other than those known to dominate their diets in summer months. In Montague Strait, Pacific herring return to overwinter in fall, and some likely remain in Montague Strait through the winter^67^. Overwintering herring could attract large adult “feeder” Chinook salmon that can be found in the northern Gulf of Alaska throughout the year^68^. Olsen et al.^48^ noted that tagged resident killer whales predominantly swam in waters deeper than 200 m in Montague Strait (though 91.9% of tag transmissions occurred between June and October). Foraging at these depths could provide access to benthic species, such as Pacific halibut, arrowtooth flounder, and sablefish, though northern resident killer whales in British Columbia have also been documented capturing Chinook salmon at depths greater than 200 m, as these fish may dive to escape predation^69^.

Hinchinbrook Entrance is a highly important area for resident killer whales in late spring and summer, at times attracting more than 150 animals^70^. This study demonstrated that Hinchinbrook Entrance had more killer whale activity earlier in the spring, especially in March and April, than previously realized^48,52^. Although killer whales have been observed in this area in substantial numbers during vessel surveys in May, June, and into July (^70^, NGOS unpublished data), during the study period, peak daily acoustic residency time for southern Alaska residents occurred in March and April, stayed high through July, and abruptly decreased in August. This active period begins earlier than spawning salmon return to Prince William Sound, which begin in late May. High resident killer whale acoustic presence also starts earlier than the spring phytoplankton bloom, which typically begins in April or May and is followed by a zooplankton bloom^71^. However, Pacific herring aggregate in Prince William Sound in March and April to spawn, especially in the northeast^67,72^. They then move relatively quickly to the entrances, where they may be feeding on seasonal blooms of *Neocalanus* copepods until late July^67^. Other small forage fish, such as capelin (*Mallotus villosus*) and Pacific sand lance (*Ammodytes personatus*), may also form feeding schools in Hinchinbrook Entrance in spring and summer. This high seasonal abundance of energetically dense forage fish may attract larger salmonids, which feed prior to moving into spawning streams, and, in turn, provide a feeding opportunity for resident killer whales in Hinchinbrook Entrance.

In Resurrection Bay, peak acoustic presence of resident killer whales occurred in May and June, coinciding with local Chinook and chum salmon abundance. Most prey samples that have been collected from foraging resident killer whales during this time demonstrate primary consumption of Chinook salmon inside Resurrection Bay^45^, and preliminary results from fecal samples illustrate predation on both chum and Chinook salmon (NGOS unpublished data). Similarly, southern resident killer whales arrive in British Columbian waters concurrently with Chinook salmon returns^73^. In this study, there was a high percentage of days per month with killer whale detections in October, but it was not matched with high daily acoustic residency times. This may suggest that resident killer whales were primarily passing through the area, rather than remaining to feed or socialize, possibly to move further into Resurrection Bay past a headland that blocked the listening range of the hydrophone. This pattern is consistent with previous vessel survey observations from October in Resurrection Bay (NGOS unpublished data).

In addition to foraging opportunities, social factors may also drive high acoustic presence and residency periods for resident killer whales at each location. Resident killer whales are highly social mammals, and the presence of conspecifics may attract additional animals, especially when prey is abundant^70^. These periods of aggregation may be of importance in establishing long-lasting social bonds among groups within the population, as well as providing mating opportunities with distantly related whales^70,74^. In contrast, during periods of relative prey scarcity, different pods within the southern Alaska resident killer whale population have demonstrated patterns of sequential habitat use, which may serve to reduce intergroup competition^75^.

In Hinchinbrook Entrance, high acoustic presence and residency by southern Alaska residents from March to July coincided with the period of highest acoustic presence and residency for the GOA transients. The majority of all GOA transient detections in this study occurred in Hinchinbrook Entrance during the spring, during which time GOA transients were recorded vocalizing for up to 6.25 h per day. If, like other transient populations, the GOA transients vocalize most often during and after kills^32–34^, then the extended bouts of vocalization observed at Hinchinbrook Entrance in spring suggest successful foraging. GOA transients in Prince William Sound and Kenai Fjords have been observed to prey most frequently on Steller sea lions (*Eumetopias jubatas*), though observations of predation on Dall’s porpoise have increased in recent years, including in Hinchinbrook Entrance in spring (^45,76^, NGOS unpublished data). Steller sea lions aggregate to forage on spawning herring schools in northeastern Prince William Sound in spring^72,77^. Dall’s porpoise show seasonal distribution patterns that coincide with patterns of herring movement in and around Prince William Sound, and their activity center shifts to the eastern Sound and Hinchinbrook Entrance in spring^78^. However, observations of GOA transient predation to date were biased to nearshore areas, and data were collected primarily from May to August. Other potential marine mammal prey likely to be available to transient killer whales in the Hinchinbrook Entrance area in spring and summer include harbor porpoises (*Phocoena phocoena*), humpback (*Megaptera novaeangliae*), minke (*Balaenoptera acutorostrata*), and gray (*Eschrichtius robustus*) whales, harbor seals, and sea otters (*Enhydra lutris*). Other transient killer whale populations in the North Pacific show seasonal patterns in dietary preference, e.g., transients in the Aleutian Islands preferentially prey on migrating gray whales in May and June and consume a variety of species in later months^40^.

The AT1 transient killer whales were detected most often in Montague Strait. AT1 transients forage primarily on harbor seals (*Phoca vitulina*) and Dall’s porpoise (*Phocoenoides dalli*)^9,45^, both of which are abundant in Montague Strait^78–80^. In recent years, there have been indications that the AT1 transients appear to have shifted from their historic range, including Montague Strait, to spend more time foraging near tidewater glaciers where harbor seals are abundant^45^. This study indicates that these transients may still spend significant time across their historic range.

The cryptic foraging strategies of transient killer whales make them difficult to visually detect in vessel surveys. Although they are also silent much of the time, this study demonstrated the utility of passive acoustic monitoring in tracking these infrequently sighted populations. For instance, during a 27-year vessel-survey study in Prince William Sound and Kenai Fjords, AT1 transients were encountered on 203 occasions and GOA transients were encountered 91 times out of 2862 survey days^49^. In contrast, in this study the AT1 transients were acoustically detected on 56 days and the GOA transients on 41 days in one year. This study also provides the first description of discrete calls produced by GOA transient killer whales that could be used for passive acoustic monitoring.

This study reinforced the utility of passive acoustic monitoring for year-round tracking of killer whales. Although it was not possible to demonstrate whether killer whales were equally likely to vocalize across seasons, if animals were vocalizing within detection range of the hydrophone, the likelihood of detecting them on different duty cycles was highly comparable throughout the year. Additionally, in areas that are difficult to access for hydrophone servicing, a shorter duty cycle, e.g., 2 min on 18 min off, could be expected to capture more than 80% of killer whale acoustic presence compared to the duty cycles used in this study. However, there were differences in the likelihood of detection for different populations. Reducing the duty cycle led to greater loss of detections of AT1 transient presence than southern Alaska resident or GOA transient presence. This is consistent with Riera et al.^81^, who found that moving from a 2/3 to 1/3 duty cycle led to greater loss of detections of West Coast transient presence than northern and southern resident presence. However, it was a novel finding that the acoustic detection rate of GOA transients was comparable to the rate for southern Alaska residents in this region. Transient killer whales are largely silent, but documented calling rates for AT1 transients are approximately five times the average calling rate measured for the West Coast transient population^33,34^. Approximately 27% more recordings of GOA transients included vocalizations in the first half compared to those of AT1 transients, suggesting that either more animals were vocalizing when GOA transients were detected or that GOA transient calling rates are even higher.

Although patterns were consistent across years when available, this study represents only 3.5 years of acoustic data. Additional years will be required to demonstrate consistency over time and to detail changes and variability in killer whale movements and residency times. We also did not estimate the listening range of the hydrophones or account for varying anthropogenic and environmental noise that may have masked killer whale vocalizations. Estimating listening range requires assumptions about mean sound source level, call frequency, and calling depth, as well as information regarding bathymetry, substrate, and sea state, and can vary substantially throughout the year (e.g.,^62^). This is important when evaluating the total number of calls recorded, but for the purposes of this study, we required only a single call to be detected in a day to assess seasonal acoustic presence, or a single call in a recording to assess daily acoustic residency. Although sound pressure level, frequency range, and active space of resident killer whale vocalizations have been estimated^28,82^, correcting for masking and detection range in this study would require an accurate measure of variance for sound source level, call frequency, and calling depth for all three killer whale populations detected in this study. This information is unavailable and logistically implausible to obtain. It is therefore not meaningful to normalize by detection area when reporting daily acoustic presence and residency time^63^. For the same reason, we did not include covariates such as wind and vessel noise. These noise sources vary seasonally in the study area, so including them as covariates may have produced spurious correlations. Additionally, killer whales have been documented to increase the amplitude of stereotyped calls commensurate with increased background noise level^83^, meaning that increased noise may not have decreased probability of detection in this study.

Finally, understanding important habitat areas for killer whales is fundamental to developing effective management policies. The Marine Mammal Protection Act requires all federally permitted human activities in U.S. waters to minimize disturbance to killer whales. Olsen et al.^48^ noted that protected status should be considered for some areas in Prince William Sound and Kenai Fjords due to their importance for killer whales. This study supports that conclusion and provides detail on high acoustic presence and residency times and locations throughout the year to inform potential seasonal management measures. For example, vessel noise beyond a certain threshold is likely to impair foraging by killer whales, while vessel speed limits can reduce noise and likely improve foraging conditions^84–86^. Seasonal vessel speed limits may therefore be appropriate in high killer whale use areas and periods, such as Hinchinbrook Entrance in spring. Notably, oil tanker lanes to Valdez, Alaska, pass through Hinchinbrook Entrance.

Attention should also be paid to the risk of environmental contamination. In particular, one stochastic, catastrophic event located in important killer whale habitat carries a high risk of population decline^50,56^. Following the *Exxon Valdez* oil spill in 1989, the AT1 transients lost 9 members (41%) and are now expected to become extinct^50^. The AB pod of the southern Alaska residents lost 13 members (33%) following the *Exxon Valdez* oil spill and has not recovered at a growth rate comparable to that experienced by the rest of the southern Alaska resident population^45,50,87^. As long-lived, slow-to-reproduce mammals that rely on matriarchal leadership, acute negative anthropogenic impacts may cause long-term injuries to killer whales beyond observable chronic direct effects, especially if females are lost^45,50,87^. As top predators, killer whales are also at particular risk of negative health impacts, as contaminants biomagnify with increasing trophic level^88,89^. Although none were of the magnitude of the 1989 spill, repeated oil spills have occurred in Prince William Sound since 1989 (e.g.,^90^). This study points to multiple high use times and areas in which a similar catastrophic event could prove devastating to killer whales.

## Methods

### Study area

We deployed Ocean Instruments SoundTrap hydrophones models ST300 STD and ST500 STD (frequency range for both models 20 Hz to 60 kHz ± 3 dB) in three locations in the northern GOA (Fig. 1). One was located in the entrance to Resurrection Bay, Kenai Fjords and one was located in each of the primary entrances to Prince William Sound: Montague Strait and Hinchinbrook Entrance. Previous vessel survey and satellite telemetry studies have shown that killer whales frequent these areas during summer months^3,9,46,48,49,51,52^.

Hydrophones were deployed in Montague Strait and Hinchinbrook Entrance from October 2016 to May 2020 and in Resurrection Bay from June 2018 to May 2020. The hydrophone in Montague Strait was first placed in Hanning Bay and subsequently moved to Little Bay (21.5 km away) in September 2017 due to high ambient noise and difficulty of retrieval in Hanning Bay (Fig. 1). The hydrophone in Hinchinbrook Entrance was first placed in Port Etches, but moved to Zaikof Bay (18.2 km away) in October 2018 also due to high ambient noise and difficulty of retrieval in Port Etches (Fig. 1). It was not possible to compare detection rates across sites because the recording periods at Hanning Bay/Little Bay and Port Etches/Zaikof Bay did not overlap.

### Data collection and field methods

The hydrophones were deployed at depths of 30.5 to 40 m on primarily gravel seafloor and were suspended approximately 3 m from the seafloor using a plastic buoy. Hydrophones were serviced once or twice per year; in some instances, the batteries were exhausted or memory capacity filled before the hydrophone was serviced. Calendar months in which a hydrophone was operating for less than half of the month were not included in this study. The hydrophones were set to record on duty cycles of 5 min on, 10 min off (5/15 min); 4 min on, 16 min off (4/20 min); or 4 min on, 11 min off (4/15 min), all with a sampling rate of 24 kHz. Deployment dates and duty cycles varied slightly among locations (Table 2).

**Table 2:** Location, deployment number and site, start and end date, duty cycle, and recording days of passive acoustic monitoring data collected in the northern Gulf of Alaska, October 2016 to May 2020.

Location	Deployment number, site	Start date (yyyy-mm-dd)	End date (yyyy-mm-dd)	Duty cycle (min on/ cycle duration)	Recording days
Montague Strait	1, Hanning Bay	2016-10-01	2017-01-31	4/20	1092
	2, Little Bay	2017-09-14	2018-05-07	4/20	
	3, Little Bay	2018-05-15	2018-09-17	4/20	
	4, Little Bay	2018-10-01	2019-09-24	5/15	
	5, Little Bay	2019-09-27	2020-05-31	5/15	
Hinchinbrook Entrance	1, Port Etches	2016-10-01	2017-06-16	4/20	1186
	2, Port Etches	2017-09-07	2018-05-12	4/20	
	3, Port Etches	2018-05-15	2018-09-30	4/20	
	4, Zaikof Bay	2018-10-01	2019-07-25	4/15	
	5, Zaikof Bay	2019-10-01	2020-05-29	5/15	
Resurrection Bay	1	2018-06-07	2018-10-02	5/15	725
	2	2018-10-02	2019-05-27	4/15	
	3	2019-05-27	2019-09-22	4/20	
	4	2019-09-22	2020-05-31	4/20	
Total					3003

### Killer whale detection and classification

Recordings were processed using the whistle and moan detector in the passive acoustic monitoring software PAMGuard (version 1.15.17) to automatically identify likely cetacean vocalizations^91^. The PAMGuard detector was tuned to prioritize sensitivity over accuracy or specificity (i.e., increased false positive rate, decreased false negative rate^92^ to minimize the probability of missing killer whale encounters. Recordings with PAMGuard detections were then aurally and visually inspected in Audacity® (version 2.3.2^93^), fast Fourier transform size 1024 with a Hann window) to confirm presence or absence of killer whale pulsed calls and/or whistles. Then, the number of days per month that killer whales were detected at each location was quantified. Values for each month were pooled across years, and a binomial logistic regression was used to identify which months had statistically high and low percentages of killer whale detections relative to the mean for each location. All statistical analyses were completed using the software program R (version 4.0.2,^94^ and the lme4 package^95^). Figures were created using the ggplot2 package^96^ in R and mapping was done using the raster package^97^ in R with elevation data from GEBCO Compilation Group^98^.

**Figure 6:**
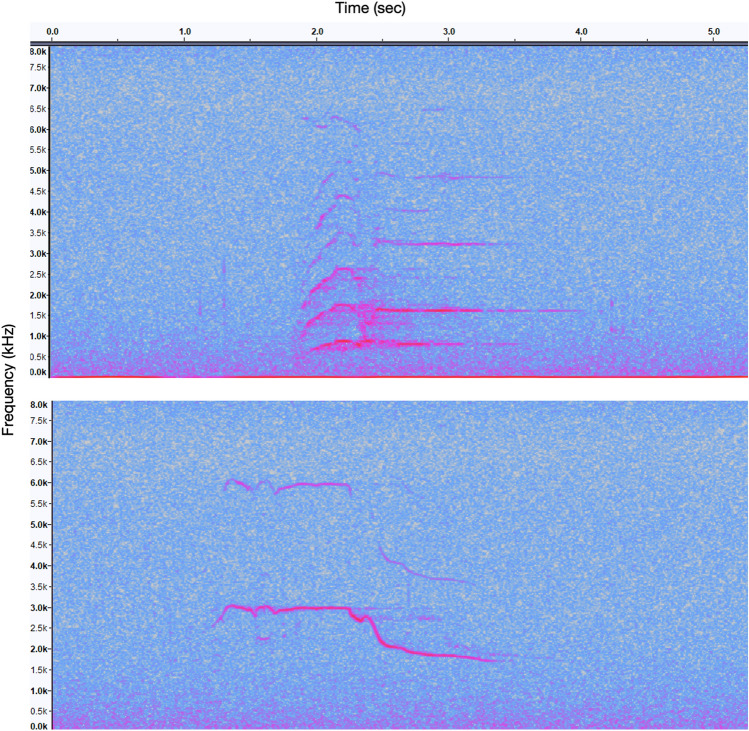
Example spectrograms of two discrete call types produced by Gulf of Alaska transient killer whales, GOA11 (**a**) and GOA12 (**b**) (fast Fourier transform size 1024, Hann window).

One year of data, June 2019 to May 2020, was further analyzed to identify which killer whale population(s) was/were present in each recording. An experienced analyst (H. Myers) classified pulsed calls as southern Alaska residents, GOA transients, AT1 transients, offshores, or unknown. Previous studies have verified that even minimally trained observers can reliably classify discrete calls^37,42^. Recordings with only whistles were classified as unknown. Southern Alaska resident killer whale calls were identified using published call catalogues^37,75^. Additional discrete calls were matched to acoustic recordings made during previous vessel surveys when resident killer whales were concurrently photographed. Calls made by the AT1 transient subpopulation were identified using the call catalogue from Saulitis et al.^34^. We first identified the two discrete calls used to identify the GOA transients (Fig. 6) in passive acoustic recordings and noted that they were distinct from southern Alaska resident or AT1 transient calls documented in published call catalogues^34,37,75^ or previously observed in field recordings. These calls also contained the audible quavering of fundamental sound frequencies and low number of different call syllables characteristic of transient killer whale calls^43^. We were then able to confirm that these calls belonged to GOA transients due to a field recording obtained during a vessel survey encounter with photographically identified GOA transients in which the same discrete calls were produced. Saulitis et al.^34^ provided 10 tentative classifications for Gulf of Alaska transient killer whale calls, but at least one of the discrete calls identified in this study was unique and it was not possible to verify whether the other discrete call matched a previously classified type. We therefore labeled these calls GOA11 and GOA12. It is not yet known how many animals within the GOA transient population use this group of calls. Offshore killer whale calls were matched to discrete calls in field recordings from vessel survey encounters in which offshore killer whales were photographically identified (J. Pilkington pers. comm., NGOS unpublished data). Unknown vocalizations consisted primarily of whistles and variable calls that were not accompanied by discrete calls, or of calls that were too faint or masked by too much noise to be identifiable.

The number of hours per day that each killer whale population was detected at each location was also described for data from June 2019 to May 2020. Hours were defined as the total number of recordings with killer whale detections divided by four for periods that recorded on a 5/15 min or 4/15 min duty cycle or divided by three for periods that recorded on a 4/20 min duty cycle.

To assess how data from hydrophones operating on different duty cycles should be compared, we identified whether vocalizations were present in the first half of each recording with killer whale detections from June 2019 to May 2020. The percentage of recordings with vocalizations in the first half of the recording was then compared across all duty cycles, locations, and seasons. This process also enabled us to identify whether potential changes in killer whale vocal behavior by season may impact our likelihood of detection. A binomial logistic regression was used to describe whether there was a difference in likelihood of detecting killer whales in the first half of the recording by month.

### Ethics statement

Passive acoustic monitoring and vessel surveys took place under National Marine Fisheries Service research permit #20341, primary permit holder Craig Matkin. The Prince William Sound Science Center (PWSSC) and University of Alaska Fairbanks (UAF) Institutional Animal Care and Use Committees (IACUC) approved the research protocols (PWSSC IACUC #2017-03-01, UAF IACUC #1492735) under which this research was conducted. All research was performed in accordance with relevant guidelines and regulations.

## Data Availability

The passive acoustic monitoring dataset is available at https://seamap.env.duke.edu/dataset/2158.
